# Technological resources during the early dissemination of the smallpox vaccine in Europe and the Americas*

**DOI:** 10.1590/S0104-59702026000100036en

**Published:** 2026-10-26

**Authors:** José Luis Duro-Torrijos

**Affiliations:** i Research and teaching coordinator, Hospital Universitario del Vinalopó. Professor of health sciences, Universidad Europea de Valencia, Alicante Campus. Elche – Alicante – Spain. jlduro@vinaloposalud.com

**Keywords:** Smallpox vaccine, Vaccination, Science, technology, and society

## Abstract

The history of smallpox vaccination demonstrates medical progress and international cooperation in the face of infectious diseases. This study examines the technological and logistical resources that enabled the preservation and transport of vaccine material between Europe and the Americas at the dawn of the nineteenth century. Drawing on primary and secondary sources, the study reconstructs the methods used to preserve the smallpox vaccine and the institutional networks that overcame technical and environmental challenges, offering a material and sociotechnical perspective that has been little explored in traditional historiography.

Smallpox was a turning point in the history of immunization, becoming the first disease to be eradicated with a scientifically validated vaccine.

Smallpox has been known since antiquity; for centuries, it caused the worst epidemics humanity has ever endured. Throughout its spread as a scourge on mankind, it spared neither age nor social class.

Estimates have highlighted its significant impact on population morbidity and mortality in both Europe and the Americas. Data place its fatality rate between 30% and 60%, giving rise to the saying that “no child belonged to their mother until they had survived smallpox” (Balaguer, Perigüell, Ballester, 2003, p.7).

Despite extensive historiographical analysis of the smallpox vaccine in Spain, which often focuses on political, scientific, or social contexts, few studies have comprehensively examined the technological and logistical aspects that enabled the preservation and distribution of the vaccine matter as a unified system. This study aims to fill that gap by analyzing the strategies used to preserve and transport the smallpox vaccine during the 18th and 19th centuries. It will specifically highlight the roles of government institutions, medical societies, and the press in disseminating scientific knowledge.

There is an extensive body of historiography analyzing the fight against smallpox in Spain. These studies focus particularly from 1799 to 1806, incorporating research that investigate on the historical, scientific, and public health contexts surrounding the introduction and dissemination of the vaccine at the Spanish court (Rigau-Pérez, Riera Blanco, 1992; Olagüe de Ros, 1995, 2008, 2011; Olagüe de Ros, Astrain Gallart, 2004; Duro Torrijos, 2014; Riera Palmero, 2015). Publications detailing the organization and execution of the Philanthropic Vaccine Expedition (1803-1813) and the events that took place in Spanish America are highlighted (Castillo y Domper, 1912; Díaz de Yraola, 1948; Fernández del Castillo, 1960; Aceves Pastrana, Morales Comes, 1997; Ramírez Martín, 1999; Tuells, Ramírez Martín, 2003; Balaguer, Perigüell, Ballester, 2003; Tuells, Duro-Torrijos, 2015; Ramírez Martín, 2021; Pérez Pérez, Vallejo, 2023; Hernández Aguilar, 2025).

## A novel preventive approach and its earliest references in Spain

Throughout the 18th century, Enlightenment thought promoted an approach based on reason and observation, enabling the development of vaccination as a strategy for preventing infectious diseases.

In this context, the scientific institution of Edward Jenner (1749-1823), an English physician, is recognized as a pioneer of smallpox vaccination. He observed that the pustules caused by cowpox, a disease that affects cattle, provided immunity to those who milked these animals (Raja, Ashwinth Jothy, 2024).

On May 14, 1796, his observations led to the first inoculation of 9 year-old James Phipps. He was injected with fluid taken from vesicles on the hand of a milkmaid named Sarah Nelmes, who had developed them after milking cows infected with cowpox. After the inoculation, the boy did not contract smallpox, even upon subsequent exposure. This demonstrated that this vaccine gave protection against the dreaded human smallpox (Viñes, 2008; Riedel, 2005).

In 1798, Jenner published his findings in the work *An inquiry into the causes and effects of the variolae vaccinae* (Jenner, 1798). He presented a new preventive method against smallpox that revolutionized the medical practice of the time and marked the emergence of vaccinology as a science (Tuells, 2012).

The Jennerian method was practiced in London, quickly spreading to major European cities between 1799 and 1800. The vaccine’s journey continued toward the Americas and territories in the East.

Just ten months after the publication of Jenner’s results, on March 21, 1799, the Spanish press was already reporting on the discovery:

In England, the physician Edward Jenner has just published a work titled *An inquiry into the causes and effects of the variolae vaccinae*, which contains a highly significant discovery highlighting the theory of inoculable diseases, while simultaneously offering a means to protect humankind from this scourge... Through numerous observations and well-conducted experiments involving specific individuals whom he names, the author has demonstrated that anyone who has contracted this disease [cowpox] acquired from a cow rather than a sick horse, as sometimes happens, remains forever immune to natural smallpox and free from the risk of contracting it, whether through contagion or inoculation (Seminario…, 21 mar. 1799, p.185).

In February 1800, the same channel of communication reported on the spread of vaccination techniques across various European cities, while simultaneously encouraging their implementation in Spain:

Experience in England has strongly supported these views, and thousands of such inoculations have been performed with great care, with results that have fully satisfied the public. This inoculation has such high confidence that it is preferred by the upper classes over the smallpox inoculation. Similar successful experiments have also been performed in Vienna and Geneva.

A society of French philanthropists intends to repeat these experiments at its own expense by importing the vaccine matter from London.

We hope that someone in Spain will follow such a beneficial example (Noticias…, fev. 1800, p.221).

In this context, Francisco Piguillem y Verdaguer (1770-1826), a physician and academic from Barcelona, began his practice on December 3, 1800, in Puigcerdà (Catalonia). The cowpox matter was sent from Paris by François Colon (1764-1812), facilitated by the ongoing contact between the Catalan and French medical communities.

The capital of the kingdom had to await a new shipment from Paris after an initial failed attempt using vaccine matter sent from Catalonia. On April 22, 1801, the scarifications were performed in Aranjuez by Ignacio de Jáuregui, a physician to the Royal Family. This procedure was subsequently implemented a month later in Madrid by Ignacio María Ruiz de Luzuriaga (1763-1822), a prominent physician and secretary of the Royal Academy of Medicine of Madrid. Following this, he engaged in intensive vaccination efforts, establishing a national network of correspondents. He sent them instructions on the practice and the innovative preventive method. In return, they provided reports on their experiences, results, and local progress with the vaccination process (Tuells, Duro Torrijos, Díaz Delgado Peña, 2012; Duro Torrijos, 2014; Duro Torrijos, Tuells, 2019).

A third hub of active vaccinators was identified in the Basque-Navarre region. Its most prominent figures included Lope de Mazarredo (1769-1820) in Bilbao, surgeons Salvador Bonor and Vicente Lubet in San Sebastián, and Diego de Bances and Vicente Martínez in Navarre (Olagüe de Ros, 1995, p.251-273).

Interest in promoting this new preventive vaccination measure, which is so beneficial to the population, emerged from both government institutions and the vaccinators themselves. Many were motivated by noble, selfless intentions, while others saw the practice as a source of significant economic gain and professional advancement, essentially, an easy path to fame and fortune.

Thus, it is important to highlight how medical and governmental institutions supported the promotion of the vaccine in Spain, while also facing technical and logistical challenges. The preservation of the vaccine fluid and its transport over long distances without loss of efficacy were among the primary obstacles. To ensure vaccine delivery to remote areas, various preservation methods were explored, including arm-to-arm inoculation, storage in glass containers, and use of vials designed to maintain the vaccine fluid under optimal conditions (Ramirez Martín, 1999).

## Obtaining and maintaining the vaccine fluid

Obtaining and maintaining the vaccine fluid in the years following Edward Jenner’s discovery of the smallpox vaccine was a complex process fraught with uncertainty. The dissemination of this preventive measure was not without controversy, stemming from both challenges regarding its acceptance or rejection and technical issues concerning the methods used to preserve and transport the vaccine fluid. Also, there were questions regarding its efficacy and the durability of the protection it offered against smallpox, specifically, whether the protection was temporary or absolute, and whether the health benefits were limited to smallpox or extended to the prevention of other diseases.

There were also concerns among the proponents focused primarily on securing and maintaining a local supply of the vaccine, with the fear that the vaccine material might run out. Spain relied heavily on European sources for the vaccine, as vaccination had already become widespread there since the late 18th century.

Cowpox was known in England, Holstein (Germany), and Lombardy. However, it was unknown in France, Spain, or the Americas. This situation sparked a race to locate cows affected by cowpox and even to test vaccination using material obtained from other animals suffering from similar forms of pox, such as horses (horse-pox) or sheep (sheep-pox) (Tuells, Duro Torrijos, 2013a).

This concern was raised both by government institutions eager to promote a preventive measure beneficial to the population and by the vaccinators driven by noble, selfless motives, as opposed to others who viewed vaccination merely as a business opportunity.

The Italian physician Luigi Sacco (1769-1836) was one example of a vaccinator deeply committed to the Jennerian cause. In 1800, he began administering the vaccine in the Cisalpine Republic (Lombardy) after discovering a cow with cowpox in neighboring Switzerland (Tuells, Duro Torrijos, 2013a).

Sacco vaccinated more than eight thousand children in Milan and organized a highly effective vaccination network. This led to his appointment in 1801 as director of Vaccination for the Cisalpine Republic and Turin, and by 1803, a Vaccination Committee had been established (García Cáceres, 2003; Olagüe de Ros, Astrain Gallart, 2004). Spain, well aware of these developments, also joined the search for vaccine material.

Due to geographic proximity, France acted as the primary supplier of the vaccine. Encouraged by promising news regarding its discovery in Italian cows, interest grew in finding a local source (Olagüe de Ros, 1995). These efforts were driven by government authorities and the vaccinators, such as Ruiz de Luzuriaga, as evidenced by an analysis of the correspondence and documentation regarding the introduction of the vaccine in Spain (Duro Torrijos, 2014).

This is the result of my observations and those of my friends, although the process of ‘yennerization’ will continue to yield new anomalies and results. I have directed my inquiries across the peninsula to determine whether ‘yennerine’ in cows was known in any province (Papeles sobre la vacuna, 1802, fol. 326).

In his voluminous correspondence, Ruiz de Luzuriaga gathered several examples attesting to this, such as a letter dated September 7, 1801, sent to the Academy’s secretary from Azpeitia (in the province of Guipúzcoa) by Javier de Murgartegui, who noted: “No one knows anything from practical experience, nor are the local cow’s familiar with this disease, according to some farmers I have asked” (Papeles sobre la vacuna, 1801a, fol. 257).

In correspondence from Soria dated November 9, 1801, the medical practitioner Josef Ubis informed Luzuriaga that:

Regarding the observations on whether the animals here contract a tumor (*gabarro*), and whether, consequently, the cows are milked, no one has yet provided any substantiated information; I have certainly made inquiries, and should I be able to gain any knowledge on the subject, I shall endeavor to do so with the utmost care and as promptly as possible, for there is no local tradition regarding this matter (Papeles de la vacuna, 1801, fol. 214).

A letter sent from Álava on November 26, 1801, by Manuel Álvarez Terrero, indicated that although similar pustules had been identified in the examined cattle, they should not be associated with the long-sought-after cowpox.

Based on the various reports I have gathered, which the local people corroborate, there is no doubt that scabs often form on the teats of breeding cows; these are mostly white and red, greatly impair the animals’ condition, and result in reduced milk yield during the eruption.

I understand, and have been assured, that this condition is never transmitted to the milkers; consequently, it cannot cause any of the ailments you mention, let alone provide immunity against smallpox (Papeles sobre la vacuna, 1801b, fol. 116).

These investigations also extended to Cuba, as evidenced by a letter sent from Havana on April 19, 1802, by the prominent physician and vaccine advocate Tomás Romay Chacón (1764-1849). Romay is described as a tireless philanthropist and a brilliant figure in the scientific and social culture of the late 18th and early 19th centuries; he dedicated himself earnestly to the fight against the era’s most significant infectious diseases, such as yellow fever (López Sánchez, 1950). However, the feat that immortalized him was the introduction and dissemination of the Jennerian vaccine in Cuba, a process that saw him leave the comforts of his home to travel into the island’s interior in search of the coveted virus and risk the lives of his children, whom he vaccinated as test subjects to overcome the fears of the general population (Diez Cabrera, Toledo Curbelo, 1997).

The letter began the text, thanking Ruiz de Luzuriaga for mediating his appointment as corresponding partner of the Academy, to continue detailing his outstanding work as introducer and disseminator of the vaccine on the island, highlighting, at this point, the references obtained regarding the possible location of native bovine fluid, a fact that he was preparing to verify: “I have hopes of obtaining it on this Island, since a resident of an inland town, where a lot of cattle are raised, He assures me that he had observed those grains on the cows, and that they used to stick them to those who milked them; but he did not notice nor did he know that it would have been noticed if they had been given to these people” (Papeles sobre la vacuna, 1802, fol. 119).

Romay’s interest in the spread and distribution of the vaccine on the island is evident in his publication called *Memoria sobre la introducción y progresos de la vacuna en la isla de Cuba* (Report on the introduction and progress of the vaccine on the island of Cuba) (Romay, 1805). He acknowledged how the benefits resulting from vaccinations carried out in Europe and the newly formed United States had reached the island via the press.

Romay and other notable figures in Havana initiated the search for the vaccine fluid. An agreement was established with the Royal Economic Society of Havana and the Economic Board of the Royal Consulate. They announced a reward of three hundred pesos to anyone who could bring the cowpox virus from other countries and four hundred pesos for anyone who discovered it in local cattle (Romay, 1805). On February 3, 1803, the periodical press published an article calling for anyone who found infected cows on local estates (Hernández Aguilar, 2025).

Although farm owners confirmed the presence of cowpox in the region’s cattle, no one claimed the prize, as Romay (1805) noted.

Interest in finding a source of vaccine lymph within the Americas was evident during the preparations for the Royal Philanthropic Vaccine Expedition (1803-1810). Several proposals were discussed, including one by Francisco Xavier Balmis (1753-1819), which was ultimately chosen and will be examined in detail in another chapter of this monograph. Additionally, there was a proposal by José Felipe Flores, dated February 28, 1803, which highlighted the following:

Given the devastation caused by smallpox in the Americas, vaccination is strongly recommended; however, it has not yet been implemented in Guatemala because cowpox has not been found there, and the bovine matter, transported between two glass plates, arrived in Havana and Veracruz, where it had been urgently requested.

… A tireless search for cowpox was conducted, and animals were examined everywhere. Unable to find the virus, Dr. Córdoba devised novel experiments involving the inoculation of sheep, based on a principle of perfect analogy… Inspections of cows continued, sheep inoculations were repeated, and requests for the virus were sent out in all directions (Díaz de Yraola, 1948, p.127).

In this same context, we find the measures taken by the Viceroy of New Spain, José Yturrigaray, who established a commission, one that has received little scholarly attention, under the direction of Félix Garda y Ferraris. This is attested to by the notary of the town of Toluca on March 22, 1803, who noted that “The Surgeon of the New Spain Infantry Regiment, commissioned by the authority of His Excellency the Viceroy..., has carried out an assessment across all the farms in the jurisdiction to inspect for cowpox” (Documentos..., 1803-1811, fol. 152).

Between March and September 1803, the physician carried out his duties but failed to find infected cows and doubted the virus’s presence in the territory of New Spain. Consequently, he requested permission to travel to the United States but rejected in late September by Viceroy Iturrigaray (Hernández Aguilar, 2025).

In early September, the Royal Decree issued by King Charles IV was announced, detailing the organization of the expedition led by Balmis to disseminate the smallpox vaccine (Tuells, Duro Torrijos, 2013b; Hernández Aguilar, 2025).

During the expedition, Balmis and his assistant, Gutiérrez Robredo, believed they had found it while passing through New Spain, specifically in the Valley of Atlixco and in Valladolid de Michoacán, in late 1804. However, after being commissioned once again six years later, Balmis acknowledged its absence and even the deception involved: “He discovered that this was a ruse by a certain scoundrel who, to pocket the 12-peso prize offered by the Bishop of Puebla to whoever discovered it, had introduced it into the cow by some unknown means; yet, based on extensive and sustained experience, he asserts that said cowpox does not exist in the local cows” (Tuells, Duro Torrijos, 2013b, p.55).

The interest in obtaining a local supply of vaccine fluid emerged from government circles, as reflected in the letter written by Secretary of State Pedro Ceballos y Guerra to the Bishop of Ourense on September 6, 1803:

Given the utmost importance of determining whether there are cows in Spain possessing bovine fluid or serum of the same nature as those in the counties of Gloucester, which have hitherto supplied Europe, as no such source had been found elsewhere, and having received reports suggesting a strong probability that cows in the Golden Valleys and Cuadramón, localities within that Bishopric, yield a bovine fluid similar to that of the aforementioned counties, and observing, as is the case there, that milkers do not contract human smallpox even if the vaccine is spontaneously inoculated through a crack in their hands, the King, having been apprised of all the foregoing, has ordered me to communicate this information (Carta secretario..., 1803, fol. 252).

The aspirations did not obtain the desired outcome, but they reflected the institution’s interest and commitment to acquiring and preserving the vaccine fluid. Consequently, early vaccinators, faced with the unsuccessful search for a local source of the cowpox virus, relied on an external supply of lymph. They focused instead on technological developments, medical techniques, and tools for the safe transport of the vaccine fluid. These methods were progressively adapted after the initial attempts to introduce the vaccine matter into hot regions failed, as the climate caused the fluid to deteriorate and lose its infectious potency (Hernández Aguilar, 2025).

## Strategies for the transport and safe mobility of the smallpox vaccine

In addition to efforts to source vaccine lymph locally to ensure a steady supply, the proper procurement method was also crucial. The entire success of vaccinations depended directly on how the vaccine material was obtained.

In this context, there was a proliferation of treatises and periodic reports that, adopting a scientific, educational approach, described the necessary resources and illustrated the various clinical stages of the pustule or vesicle, indicating the right moment to perform the arm-to-arm procedure. A prime example is a text published in 1801 by the physician Pedro Hernández (1801, p.41-42), which had a tremendous impact and wide circulation at the time:

Since the success of the procedure depends primarily on the fluid introduced beneath the epidermis, it is essential to select the moment when that fluid has reached the degree of perfection and maturity required to effectively transmit the disease. The most favorable time is between the seventh and eleventh day of the illness; before this stage, the vesicle is not sufficiently developed, and the fluid it contains is neither fully elaborated nor perfected.

Once these initial challenges were overcome, the preservation and transport of the vaccine emerged as other critical issues associated with the Jennerian fluid.

The long distances the vaccine had to travel posed a serious problem. The fluid often arrived dried out and unusable, particularly on journeys where it was exposed to temperature fluctuations that accelerated or retarded the process, affecting its quality. Temperature was a factor frequently highlighted by medical professionals of the time. Thus, in his 1805 publication *Cartilla de vacunas* (Vaccine Handbook), Joseph Morales noted that, while vaccination could be performed at any time, one had to bear in mind that “heat somewhat accelerates the vaccine’s progress, while cold somewhat retards it” (Ramírez Martín, 1999, p.524).

Vaccinations could be performed using “fresh or dried vaccine matter” (Balmis, 1804, p.192). In the fresh one, the matter was obtained directly by lancing a vaccine pustule with the instrument used for the vaccination, drawing the fluid from a subject who had to be present. “This operation is called arm-to-arm vaccination” (p.193).

In the dried case, the vaccine fluid had to be preserved in various media, facilitating its transport. Methods included soaking cotton, using the lancet, or employing glass plates to press and preserve the vaccine fluid.

In well-described procedures, specifically the use of absorbent cotton, “the vaccine fluid dries on the absorbent cotton to the point where it cracks upon being touched, exposing the physician to the risk of administering an ineffective vaccine or one that produces no effect at all, leaving the vaccinated person exposed to the very danger of contracting smallpox” (Balmis, 1804, p.193).

Through the lancet,

dried vaccine pus on the tip of a lancet exists in two very different states. First, it commonly oxidizes the lancet, undergoing a decomposition that alters its nature and produces a ‘false vaccine,’ much like lint soaked in dried vaccine fluid, or else it fails to produce any effect at all. Second, if the lancet does not oxidize, the pus acquires a transparent, glassy consistency with sharp edges; when inserted into the skin, it acts as a foreign body, causing immediate irritation and resulting in a ‘false vaccine’ (Balmis, 1804, p.193).

In the latter case, in the use of glass,

if vaccine matter is placed between glass plates, it can retain its properties for a long time without undergoing any alteration, provided the following precautions are observed: once the circular vesicle of the pustule has been pricked across its entire surface, the exuding fluid is collected on a flat, smooth glass plate; as soon as a sufficient quantity has been gathered, a second plate of equal size is applied. When both are adequately charged with pus, the two plates are joined together with their moistened surfaces facing each other, and their edges are sealed precisely with a small amount of sealing wax. Upon arrival at the destination, the plates are easily separated by bringing a glowing coal near them; when ready to vaccinate, a drop of distilled water is placed on one of the plates and mixed thoroughly with the tip of a lancet (Balmis, 1804, p.194).

The first two options, cotton and the lancets, presented drawbacks, as the quality of the fluid, and consequently its prophylactic efficacy, could not be guaranteed (Ramírez Martín, 1999, p.531).

The use of glass plates was the safest method; indeed, through this procedure, the vaccine arrived in Spain and spread across the country.

The technique was documented in numerous treatises on the vaccine and even in the press.

One should wait until the vesicle or pustule has reached full maturity. At that point, a circular incision should be made to open as many of the vesicles composing it as possible. A piece of absorbent cotton, equal in volume to the concavity of the glass plate, should then be applied with its softest and most porous side facing the lesion, and gently pressed against the pustule from time to time with the blade of the lancet. In this way, a single pustule is sufficient to saturate the cotton thoroughly. The advantage of being able to receive fresh vaccine lymph over any distance and preserve it in the event of interruptions in vaccination is of great importance (Carta sobre..., 9 set. 1802, p.174).

To use the bovine fluid preserved by this method, the application instrument must first be wetted with cold, very clear water. The area containing the fluid should then be rubbed with the water until the fluid reaches a slightly thick consistency. After that, the lancets that will be used for the punctures should be “loaded or wetted” (Calleja, 1814, p.5-6).

The need to travel long distances to distribute the vaccine material led to experimentation with other methods, such as inoculating a healthy subject with material derived from the pulverized scabs of a smallpox patient.

The press disseminated all results and advances regarding the smallpox vaccine emerging from Europe. Many of these reports were authored by prominent pro-vaccination activists, such as Jean de Carro, a Swiss physician who was instrumental in introducing vaccination to Central Europe and the Near East.

It was previously believed that the cowpox scab could not reproduce the disease. At this point, others and I perceived a difference between smallpox and cowpox. However, an Edinburgh surgeon named Bryce has repeated numerous experiments proving that the scab, when pulverized and moistened with water, produces the vaccine just as successfully as the clearest fluid. This fact seems highly important, considering how easily the cowpox virus can be preserved within scabs and transported over long distances. Vienna, February 2, 1804. De Carro (Nuevo…, 10 maio 1804, p.304).

Building on Jean de Carro’s comments about the importance of obtaining fresh vaccine lymph, regardless of distance, and the need to preserve it if vaccinations are interrupted, the same newspaper emphasized the innovation and technological advancements represented by the small vials created in late 18th-century London by Sir Lewis, an apothecary at the Vaccine Institution (Duncan, 1801).

A simple method for transporting vaccine lymph has recently been devised in England, and it has proven highly successful in practice. Since the main objective is to prevent the lymph from being exposed to air, it is collected in a small, smooth cavity made at the end of the glass stopper of a small glass vial. The stopper extends almost to the bottom of the vial and is fitted so precisely to its neck that it forms a perfectly airtight seal. As a result, the vaccine lymph, being in contact only with the small amount of air contained within the narrow vial, neither decomposes nor evaporates. When it is to be used, the vial needs only to be opened, and a lancet, previously dipped in cold water, is passed several times over the small concavity at the tip of the stopper. The maker of these vials holds an exclusive patent and sells them in London at Parker’s premises on Fleet Street (Carta sobre…, 9 set. 1802, p.176).

It was precisely the person responsible for the newspaper’s publication, Juan Antonio Melón González (1758-1843), a clergyman, scholar, and Enlightenment writer, who sent a letter dated August 10, 1803, to the Secretary of State and of the Office, Pedro Cevallos y Guerra (1759-1839), to point out the importance of the news and the need to implement it in our country.

This letter coincided with preparations for the Royal Philanthropic Vaccine Expedition, an expedition whose primary objective was to spread the smallpox vaccine to the overseas colonies by using children as reservoirs for the fluid, a method that Melón questioned:

It is said that the expedition dispatched by order of the King to spread vaccination throughout the Americas will carry individuals in whom the vaccine will be passed successively the vaccine lymph fresh. This method is subject to contingencies that could frustrate the purpose of so beneficial an undertaking, for example, if the vaccinated individual were to fall ill or die, if the vaccination failed to take in one of them, as sometimes occurs, or if a spurious pustule developed, as is also occasionally observed. To avoid these difficulties, a new method has recently been devised in England for transporting vaccine lymph in specially designed glass vials, which are sold in London at Parker’s premises on Fleet Street. These vials have won such approval among British medical practitioners that the King has granted their inventor an exclusive patent (Carta secretario..., 1803, fol. 258).

Based on these references, Melón recommended that Cevallos have José de Anduaga y Garimberti (1751-1822), the secretary of the embassy in London, send over some small vials via the first available post so they could be replicated at the San Ildefonso glass factory. These containers would ensure the preservation of the vaccine fluid during the expedition bound for the Americas.

I am aware that those traveling to the Americas by order of the King, our lord, do not possess them; perhaps it would please His Majesty if Your Excellency were to instruct Mr. Anduaga to send one or two dozen, along with the method for collecting the fluid in them, by the first available post, so that, upon inspection, they might be manufactured at San Ildefonso and supplied not only to those bound for the Americas but also to the general public wishing to use them to disseminate such an important preventive measure (Carta secretario..., 1803, fol. 259).

A marginal note in the document records the specific request: “Ask Anduaga for the two dozen small vials, along with the method for collecting the fluid in them” (Carta secretario..., 1803, fol. 259).

In compliance with the Royal Order, Anduaga sent the requested items from London by return mail on September 3rd, along with a manual for their use.

I am sending a package containing two dozen small glass vials, purchased at Parker’s shop on Fleet Street, via the official courier Don Eugenio Gutiérrez, to transport and preserve vaccine fluid. I have been assured that no further explanation regarding their use is required beyond noting that, due to the shape of the stopper and the significant length of the section that fits inside the vial, they seal hermetically. As the fluid adheres to the inner part of the stopper, the latter serves as a convenient means to extract the desired amount simply by removing it (Carta secretario..., 1803, fol. 264).

Upon receiving the novel small vials, José Antonio Caballero (1754-1821), Secretary of State and of the Office of Grace and Justice, forwarded 12 of the 24 containers to the King’s Board of Chamber Surgeons for examination, noting that “if they merit the same acceptance and approval here that these vials have garnered in England, they could easily be replicated at the Royal Factory of San Ildefonso” (Carta secretario..., 1803, fol. 264).

They concluded that:

They appeared very useful and preferable to the glass containers previously used; since more time is required to introduce the fluid, it risks volatilizing or dissipating upon contact with the air, whereas transferring it into the small vials is a very rapid process, resulting in safer preservation and easier, more convenient transport (Carta secretario..., 1803, fol. 252).

To verify this claim, the Board decided to send six of the 12 small vials to the Colegio de San Carlos in Madrid for the collection of vaccine fluid

...from genuine vaccine recipients, placing the vaccine lymph alone in three vials and, in the others, a proportionate quantity diluted with distilled water, since previous experiments had shown that, when diluted in this manner, the pus of natural smallpox produced the same, though milder, effect as when inoculated in its pure form. If the same proves true of vaccine lymph, the spread of vaccination would be facilitated by the greater quantity of lymph that could be sent in each vial (Carta secretario..., 1803, fol. 252).

On October 21, 1803, approval was granted for the production of small vials at the Royal Factory of San Ildefonso to expand their usage.

Government institutions showed strong interest in properly preserving the vaccine fluid to ensure its safe transport. On November 29 of that year, Anduaga sent another small vial from London. This vial contained vaccine fluid prepared according to the method adopted by the issuing center: “Accompanying the vial is a printed sheet on which the institute outlines the rules for administering the vaccine, as well as a small octavo volume containing the report or account of vaccine inoculation as practiced by the institute during the years 1800, 1801, and 1802” (Carta secretario..., 1803, fol. 268).

Cevallos sent a letter on February 11, 1804, to Ruiz de Luzuriaga, who was a secretary of the Madrid Medical Academy. In the letter, he included “a small vial of the kind recently invented in England, containing the virus,” along with instructions for the Medical Academy to use it as they saw fit (Carta de Pedro Ceballos…, 1804).

Just five days later, the secretary of the academy presented it at the institution’s ordinary session:

The Secretary read the minutes of the previous meeting and reported a royal order communicated by His Excellency the Protector, dated 11 February, enclosing a small vial recently invented in England for preserving vaccine lymph according to the method adopted by the Institution for Promoting Vaccination in London. Also enclosed were a small volume in English containing the Institution’s report on vaccination, based on its practice in 1800, 1801, and 1802, and a separate sheet setting out the instructions for the procedure. The Academy resolved that these materials be forwarded to Messrs. Juan de Peñalver and Tomás García Suelto so that they might incorporate them into the report on vaccination with which they had been entrusted (Libro de actas…, 1804, fol. 321).

The initial expectations for this useful container began to diminish. Cevallos communicated to Caballero, on April 13, through the Secretary of State and the Office, that “the effects experienced in seven different individuals did not meet the hopes of the Board” (Carta de Pedro Ceballos…, 1804). This statement referred to the Board of Surgeons of the Royal Chamber and highlighted that the anticipated benefits were not realized. As a result, the idea of producing a sufficient quantity to sell in the Royal Warehouse after confirming its usefulness started to fade (Libro de actas…, 1804, fol. 322).

The King has proposed that his ambassador in London request further instructions from Dr. Jenner regarding the method for collecting vaccine lymph and placing it into specially designed vials. Additionally, he seeks clarification on whether the lymph should be collected directly from cows or from vaccinated individuals. The ambassador was also instructed to send, together with these instructions, two vials containing vaccine lymph prepared according to that method, together with a description of the procedure followed in England (Carta de Pedro Ceballos…, 1804).

The initial expectations surrounding the small English vials were dashed by the lack of results. It was the very person who had taken an interest in their use on the Peninsula, Juan Antonio Melón, editor of the *Semanario de Agricultura y Artes Dirigido a Párrocos*, who would later publish an article in that same periodical on April 12, 1804, highlighting the container’s lack of practicality and reliability.

Since the English had praised the method of transporting the vaccine fluid in specially designed small vials, the Minister of State, with highly commendable zeal, requested a supply from London. However, upon testing them in Madrid, it was found that they did not preserve the fluid well. Two factors may contribute to this: first, the stoppers of these vials do not extend far enough to fill the entire cavity, leaving behind, after sealing, sufficient air to alter the fluid; and second, since the vials are made of glass, light may have acted upon the fluid, depriving it of its potency (Concluye…, 12 abr. 1804, p.239).

A month later, on May 12, Ruiz de Luzuriaga reaffirmed this same concept, conveying to Minister Caballero the results of practical trials using the vaccine fluid sample that had been sent to the medical academy in the innovative container on February 11.

Although the surgeon Don Juan de Azaola performed several vaccinations using it, all of them failed. This undoubtedly stemmed from the small vial having been opened before reaching our hands, causing the dissipation of the hydrogen gas with which the vial is meant to be filled, a very cumbersome method and by no means preferable to the use of glass plates, on which the vaccine has been perfectly preserved in every shipment I have sent throughout the Peninsula, even though the ones I sent to Havana did not take (Carta de Pedro Ceballos…, 1804).

Ruiz de Luzuriaga confirmed the negative results reported by the Board of Royal Surgeons while defending the preservation method used until that point, specifically the use of glass plates. He had established a network of correspondents around this method, which had produced excellent results, except for the shipment sent to Tomás Romay in Havana.

During the initial stages of the vaccine’s introduction, three methods for preserving and transporting the vaccine fluid were identified: the dry method, involving the impregnation of silk or cotton with vaccine pus, followed by drying and storage; the glass method, involving the preservation and shipment of vaccine pus sealed between glass slides or inside vials. However, both methods proved inadequate for long voyages, particularly in hot climates, as the pus would deteriorate and lose its infectious potency. Consequently, the in vivo or arm-to-arm approach emerged as the safest method for propagating the vaccine. This technique demanded specific logistics, healthcare infrastructure, and institutional effort (Hernández Aguilar, 2025). A clear example of this is the preparation and execution of the Royal Philanthropic Vaccine Expedition, which we will now describe in detail.

There was significant concern about transporting the fluid, particularly to overseas territories where smallpox was still present. This situation led authorities in various viceroyalties to urge the Crown to send the new remedy as soon as possible. The Bourbon monarchy, having adopted scientific expeditions to promote progress and assert control, approved the project called the Royal Philanthropic Vaccine Expedition. Led by Francisco Javier Balmis, the mission opted to use human reservoirs to transport the vaccine, transferring it from arm to arm, creating a vital chain formed by a succession of children, most of whom were drawn from foundling homes and orphanages.

Details regarding the organization, existence, and preparation of the vaccine expedition were published in the periodical press in July 1803, such as in the report carried by the *Mercurio de España*:

The remarkable discovery of the vaccine, recognized in Spain and throughout much of Europe as an effective safeguard against smallpox, has led the King to express a paternal concern for its distribution across his territories in the Indies, where this devastating disease often claims many victims. To address this issue, His Majesty has ordered the organization of a maritime expedition, following the advice of the Council and various specialists. This expedition will consist of skilled physicians who are dedicated to the mission and will be led by Honorary Royal Physician Don Francisco Xavier de Balmis. The Royal Treasury will fund the expedition. The team plans to transport a sufficient number of children, inoculating them successively during the voyage to maintain the vaccine’s potency. Upon arrival, they will conduct the initial arm-to-arm inoculations and continue this practice throughout the Americas and, if feasible, in the Philippine Islands (Noticia de la expedición…, jul. 1803, p.382).

Balmis’s proposal relied exclusively on using children to transport the fluid. This method had been recommended by Jenner (Rusnock, 2009). As a result, the children became significantly important, a fact highlighted in the regulations and itinerary that Balmis presented to the Board of Surgeons (Castillo, Domper, 1912). The children who were inoculated with cowpox, from whom vaccine fluid could be harvested after eight to ten days once the pustule had matured, were referred to as *niños vacuníferos* (vaccine-carrying children). Immunization would proceed from one child to the next, establishing a chain of successive inoculations that enabled the vaccine to be transported.

Balmis recommended recruiting children between the ages of 5 and 8 who had not previously contracted smallpox, as this offered a degree of assurance compared to adults, who might have already had the disease. Consequently, he placed special emphasis on ensuring that the selection process involved “scrupulous verification to confirm that they have not yet suffered from natural or inoculated smallpox, nor have they been vaccinated, for all such individuals are unsuitable” (Ramírez Martínez, 2003, p.86). To ensure the procedure’s success and avoid failure, he inoculated two children in succession using multiple punctures, allowing for the development of several vaccine pustules on each child. The vaccine-carrying child was present throughout every route and stage of the Royal Philanthropic Vaccine Expedition.

The use of children for scientific purposes was a reality throughout the 18th century, driven by a utilitarian mindset and the extension of the scientific method to serve the interests of the State. The Royal Philanthropic Vaccine Expedition was a clear example of this practice, as the mission’s success depended directly on the number of children involved, their continuous supply to sustain the arm-to-arm chain, and the maintenance of their perfect health.

## Final considerations

The preservation and transportation of the vaccine fluid during the early days of smallpox vaccination had technical challenges that determined its efficacy. The preservation methods employed, from storage between glass plates and in experimental vials to propagation via arm-to-arm vaccination, revealed specific limitations under adverse weather conditions and during long journeys, driving logistical innovations that were crucial for the vaccine’s effective expansion.

The influential role of institutional networks, the periodical press, and innovations as channels for vaccination cannot be overstated. This interplay facilitated the transatlantic transfer of vaccine materials. This perspective is integral to a historiographical approach that emphasizes the material and technological networks that shaped early public health.

The reach or success of these vaccination campaigns was not without criticism. This preventive practice gave rise to uncertainty, fear, or resistance. These social reactions and the actions of government authorities are analyzed by various local entities, whose findings represent a significant contribution to the history of public health (Molina del Villar, 2017).

From a technological and scientific perspective, smallpox vaccination helped develop new methods for preserving and transporting vaccines. Interest in improving the stability of the vaccine fluid led to experimentation with various techniques, such as impregnating cotton with the vaccine, storing it in glass containers, or using hermetically sealed vials. Although some of these techniques had limitations, they laid the groundwork for the development of more sophisticated methods in the future.

Thus, obtaining and preserving the vaccine fluid was a crucial technical challenge for ensuring the effectiveness of vaccination, a task that required proper organization and the involvement of both government and scientific spheres.

In conclusion, the view expressed in December 1803 by Ruiz de Luzuriaga, secretary of the Madrid Academy of Medicine, is noteworthy. He highlighted the need to implement a methodical vaccination program by creating a commission or central board, following the examples set by France and England, tasked with monitoring cases and preventing shortages of the vaccine fluid.

This philanthropic institution relieves the government of the burden, as exists in Spain, of attending to all matters of public charity; it would feature a central commission in the capital composed of the most distinguished figures, liaising with Captains General, archbishops, bishops, and physicians and surgeons of the highest repute and merit, as well as an apothecary to supply ‘Extract of Saturn’ or other inexpensive medications that might occasionally be required during vaccinations. In this way, the vaccine lymph could be maintained throughout the Kingdom, the introduction of counterfeit vaccines prevented, and population growth promoted.

A workshop established at the glass factory to use the broken glass for shipping the vaccine fluid, certain postal exemptions for shipments sealed with the commission’s stamp, and a measure of government protection would be the true means of disseminating this healthy and beneficial discovery from the capitals to the smallest hamlet; this would make a simultaneous, widespread shortage highly unlikely and ensure the free transfer of the vaccine between commissions, supported by instructions, drafted in a catechism format, on how to administer vaccinations and avoid ineffective vaccines that fail to protect against smallpox (Informe…, dez. 1803, fol. 4).

A health-related interest emerges within a context characterized by the establishment of a new form of governance, focused on the collective of living beings organized as a population.

Viewing health as a fundamental objective of political power was a recurring concern of European Enlightenment reformism; this goal was pursued through the implementation of a health policy whose essential features included measures to control mortality, childhood issues, and birth rates, in short, the operation of medicine as an instrument of social control.

To reach all social strata, this revitalized science relied on platforms such as the periodical press and scientific publications, both playing medical and educational roles in the medicalization of the family unit.

However, despite the concerns and calls for the implementation of vaccination, the government response was lukewarm; no legislative measures were enacted to support Jenner’s invention formally. Exceptions included the issuance of Royal Orders between August and September 1803, announcing the Philanthropic Vaccine Expedition, and the Royal Decree of 1805, which mandated the establishment of vaccination clinics in hospitals within provincial capitals. These clinics were intended to provide the procedure free of charge to the poor, yet the initiative met with little acceptance among the general population.

The history of smallpox vaccination illustrates the sociopolitical and technical complexities involved in adopting biomedical innovations. In addition to scientific and technological advancements, the acceptance of vaccination demanded educational efforts, effective communication strategies, and official support. The challenges that early vaccinators encountered persist today, including vaccine resistance, misinformation, and logistical barriers to immunization distribution.

## Data Availability

Not deposited in a data repository.
