# Supplementary material for: Technological resources during the early dissemination of the smallpox vaccine in Europe and the Americas
Source: Hist Cienc Saude Manguinhos. 2026 Oct 26;33(Suppl 1):e2026036. doi: 10.1590/S0104-59702026000100036en (PMC13719173; doi:10.1590/S0104-59702026000100036en)
Supplement: Translation

# Translation: Recursos tecnológicos en el inicio de la difusión de la vacuna contra la viruela en Europa y América*

**DOI:** 10.1590/S0104-59702026000100036

**Authors:** José Luis Duro-Torrijos

**Affiliations:** i Coordinador de investigación y docencia, Hospital Universitario del Vinalopó. Profesor de ciencias de la salud, Universidad Europea de Valencia, Campus de Alicante. Elche – Alicante – España. jlduro@vinaloposalud.com

**Keywords:** Vacuna contra la viruela, Vacunación, Ciencia, tecnología y sociedad

## Abstract

La historia de la vacunación contra la viruela evidencia el progreso médico y la cooperación internacional frente a las enfermedades infecciosas. Este estudio examina los recursos tecnológicos y logísticos que permitieron la conservación y el transporte del fluido vacunal entre Europa y América en los albores del siglo XIX. A partir de fuentes primarias y secundarias, se reconstruyen los métodos de preservación de la vacuna antivariólica y las redes institucionales que permitieron sortear desafíos técnicos y ambientales, aportando una mirada material y sociotécnica escasamente estudiada en la historiografía tradicional.

La viruela marcó un punto de inflexión en la historia de la inmunización al convertirse en la primera enfermedad para la cual se desarrolló una vacuna científicamente validada con resultados exitosos.

Como enfermedad, la viruela es una conocida desde la Antigüedad, su presencia causó durante siglos las peores epidemias que ha sufrido la humanidad. Desde entonces, en su propagación como azote de la humanidad, su presencia no hacía distinción de edad ni clase social.

Estimaciones han señalado su gran incidencia desde el punto de vista de la morbilidad y mortalidad en la población, tanto en Europa como en América. Hay datos que cifran su letalidad entre un 30% y un 60%, por lo que se solía decir que “ningún niño era de su madre hasta que había superado la viruela” (Balaguer, Perigüell, Ballester, 2003, p.7).

A pesar del intenso análisis historiográfico sobre la introducción de la vacuna contra la viruela en España, enfatizado en el contexto político, científico o social, son escasos los estudios centrados de forma exhaustiva en los aspectos tecnológicos y logísticos que permitieron la conservación y perpetuación del fluido vacunal como un bloque unitario. Esa área pretende ser abordada en el presente estudio, a través del análisis de las estrategias empleadas para la conservación y el transporte de la vacuna contra la viruela en los siglos XVIII y XIX, con especial énfasis en el papel desempeñado por las instituciones gubernamentales, las sociedades médicas y la prensa en la difusión del conocimiento científico.

Existe una abultada historiografía cuyo objeto de análisis es la lucha contra la viruela en España. Esos estudios ponen especial atención al periodo comprendido entre 1799-1806 que integran textos con investigaciones que incorporan contribuciones del contexto histórico, científico y sanitario de la introducción y difusión de la vacuna en la corte española (Rigau-Pérez, Riera Blanco, 1992; Olagüe de Ros, 1995, 2008, 2011; Olagüe de Ros, Astrain Gallart, 2004; Duro Torrijos, 2014; Riera Palmero, 2015). En particular, destacan de manera especial las publicaciones sobre la configuración de la Expedición Filantrópica de la Vacuna (1803-1813) y aspectos que acaecieron en la América española, así como desarrollo de la misma (Castillo y Domper, 1912; Díaz de Yraola, 1948; Fernández del Castillo, 1960; Aceves Pastrana, Morales Cosme, 1997; Ramírez Martín, 1999; Tuells, Ramírez Martín, 2003; Balaguer, Perigüell, Ballester, 2003; Tuells, Duro-Torrijos, 2015; Ramírez Martín, 2021; Pérez Pérez, Vallejo, 2023; Hernández Aguilar, 2025).

## Una novedosa variante preventiva y sus primeras referencias en España

A lo largo del siglo XVIII, el pensamiento ilustrado impulsó un enfoque basado en la razón y la observación, lo que permitió el desarrollo de la vacunación como estrategia para la prevención de enfermedades infecciosas.

En este contexto, la intuición científica del médico inglés Edward Jenner (1749-1823) lo coloca como pionero en la vacunación contra la viruela, al observar que las pústulas de las viruelas que sufrían las vacas, enfermedad denominada *cowpox*, proporcionaban inmunidad a quien las ordeñaban (Raja, Ashwinth Jothy, 2024).

Sus observaciones propiciaron, el 14 de mayo de 1796, la primera inoculación al joven de James Phipps, de 9 años, con el fluido de las vesículas que la vaquera Sarah Nelmes había contraído en su mano al ordeñar vacas que estaban afectadas de *cowpox.* El joven, tras ser inoculado, no desarrolló la viruela ni siquiera después de serle inoculada posteriormente, demostrando que esta vacuna protegía contra las temidas viruelas humanas (Viñes, 2008; Riedel, 2005).

En 1798, Jenner daba a conocer sus resultados en su obra *An inquiry into the causes and effects of the variolae vaccinae* (Jenner, 1798). En ella exponía a la sociedad el nuevo método preventivo contra la viruela que revolucionó la práctica médica de la época, así como el despertar de la vacunología como ciencia (Tuells, 2012).

El método jenneriano se practicó en Londres, pasando rápidamente a las principales ciudades europeas entre 1799 y 1800. Un viaje de la vacuna que continuó dirección al continente americano y a los territorios orientales.

Tan solo diez meses después de la publicación de los resultados de Jenner, el 21 de marzo de 1799, la prensa española ya se hacía eco del hallazgo:

En Inglaterra acaba de publicar el Médico Eduardo Jenner una obra con el título de *Examen de las causas y efectos de las viruelas de las vacas* que contiene un descubrimiento muy particular que puede dar mucha luz a la teórica de las enfermedades que se pueden inocular, al mismo tiempo que puede ser útil para preservar de este azote al género humano… El autor de esta obra ha demostrado con muchas observaciones y experimentos bien hechos con personas cuyos nombres expresa, que todos los que hayan padecido esta enfermedad [*cowpox*] con tal que la hayan tomado, no del caballo enfermo, como sucede algunas veces, sino de la vaca, quedan para siempre exentos de las viruelas naturales y libres de padecerlas, ya sea por contagio, o por inoculación (Seminario…, 21 mar. 1799, p.185).

Meses más tarde, en febrero de 1800, el mismo canal de comunicación se hacía eco del avance de la técnica de la vacunación por las diferentes ciudades europeas, alentando, a su vez, su puesta en marcha en España:

La experiencia ha confirmado hasta ahora estas opiniones en Inglaterra, y se han hecho millares de inoculaciones de esta especie con los cuidados y las pruebas más satisfactorias para el público. Tienen allí tanta confianza en esta inoculación, que se practica entre las gentes principales con preferencia a la de las viruelas. En Viena y en Ginebra se han hecho las mismas experiencias con el mismo éxito.

Una sociedad de filántropos franceses va a repetir, a su costa, estas experiencias haciendo traer de Londres la materia que sirva para la inoculación de la Vacuna.

Deseamos que en nuestra España haya quien imite tan benéfico ejemplo (Noticias…, feb. 1800, p.221).

En este contexto, Francisco Piguillem y Verdaguer (1770-1826), médico y académico de Barcelona, inauguró su práctica el 3 de diciembre de 1800 en Puigcerdá (Cataluña). El pus vacuno fue remitido desde París por François Colon (1764-1812), gracias al contacto mantenido por la medicina catalana y la francesa.

La capital del reino, tras un primer intento fallido con vacuna remitida desde Cataluña, tuvo que esperar una nueva remesa procedente de París. Las escarificaciones se fechan el 22 de abril de 1801 en Aranjuez, por el médico de la Real Familia Ignacio de Jáuregui, implementándose un mes más tarde en Madrid por Ignacio María Ruiz de Luzuriaga (1763-1822), destacado médico y secretario de la Real Academia de Medicina de Madrid. Dicho profesional inició desde entonces una intensa actividad vacunadora en la que llegó a trazar una red nacional de corresponsales a quienes remitía instrucciones de su práctica y del novedoso método preventivo, a cambio de la notificación de sus experiencias, resultados y avancen locales del proceso vacunal (Tuells, Duro Torrijos, Díaz Delgado Peña, 2012; Duro Torrijos, 2014; Duro Torrijos, Tuells, 2019).

Se identificó un tercer núcleo de activos vacunadores en la zona Vasco-Navarro, cuyos exponentes más destacados fueron Lope de Mazarredo (1769-1820) en Bilbao, los cirujanos Salvador Bonor y Vicente Lubet en San Sebastián, y Diego de Bances y Vicente Martínez en Navarra (Olagüe de Ros, 1995, p.251-273).

El interés suscitado por propagar la reciente medida preventiva de la vacuna tan beneficiosa para la población provino tanto de las instituciones gubernativas como de los propios vacunadores, muchos de ellos con una afán noble y desprendido frente a otros, que veían en dicha operación grandes oportunidades económicas y de desarrollo profesional, en definitiva, una manera fácil de obtener fama y fortuna.

De este modo, importa resaltar cómo en España la difusión de la vacuna encontró respaldo en instituciones médicas y gubernamentales, pero también enfrentó desafíos técnicos y logísticos. Entre los principales obstáculos se encontraba la conservación del fluido vacunal y su transporte por largas distancias sin que perdiera eficacia. Para garantizar el suministro de la vacuna en territorios remotos, se exploraron diversos métodos de conservación, como la inoculación de brazo a brazo, el almacenamiento en cristales y el uso de frascos diseñados específicamente para preservar el fluido vacunal en condiciones óptimas (Ramirez Martín, 1999).

## La obtención y perpetuación del fluido vacunal

La obtención y perpetuación del fluido vacunal en los primeros años, tras el descubrimiento de la vacuna contra la viruela por Edward Jenner, fue un proceso complejo y lleno de incertidumbre. La difusión de esta medida preventiva no estuvo exenta de controversias, tanto por dificultades en su aceptación o rechazo como por cuestiones técnicas relacionadas con sus métodos de conservación y transporte del fluido vacuno. A ello se sumaban otras interrogantes sobre su eficacia y durabilidad en la protección contra la viruela, si ésta tendría una cualidad temporal o absoluta, si sus beneficios sanitarios se circunscribían sólo a la viruela o precavía otras enfermedades.

Del mismo modo, entre los propios defensores también se despertaron inquietudes encaminadas más a la obtención y perpetuación de la vacuna en un ámbito local ante el temor de que se agotara el fluido vacuno. En este sentido, en cuanto a la obtención de la vacuna, España dependió en gran medida de las fuentes europeas, donde la vacunación ya se había extendido desde finales del siglo XVIII.

El *cowpox* era conocido en Inglaterra, en Holstein (Alemania) o Lombardía, sin embargo, se desconocía en Francia, España o América. Esa situación originó una carrera para localizar vacas afectadas por el *cowpox*, incluso para ensayar la vacunación con material obtenido en otros animales que padecieran formas similares de viruelas, como los caballos (*horse-pox*) o las ovejas (*sheep-pox*) (Tuells, Duro Torrijos, 2013a).

La inquietud fue elevada, tanto por las instituciones gubernativas deseosas de propagar una medida preventiva beneficiosa para la población como de los propios vacunadores, ellos con un afán noble y desprendido frente a otros que veían en la vacunación una oportunidad de negocio.

Un ejemplo de vacunador muy comprometido con la causa jenneriana fue el médico italiano Luigi Sacco (1769-1836), que, en 1800, comenzó a vacunar en la República Cisalpina (Lombardía) tras descubrir una vaca con *cowpox* en la vecina Suiza (Tuells, Duro Torrijos, 2013a).

Sacco vacunó en Milán a más de ocho mil niños y organizó una red muy eficaz de vacunación que le supuso su nombramiento en 1801 como director de Vacunación de la República Cisalpina y Turín, llegando a contar desde 1803 con un Comité de Vacunación (García Cáceres, 2003; Olagüe de Ros, Astrain Gallart, 2004). España, que no fue ajeno a todas estas noticias, se sumó también a la búsqueda de material vacuno.

A su vez, Francia, por proximidad geográfica, actuó como principal proveedor de vacuna. Alentados por las esperanzadoras noticias sobre su hallazgo en vacas italianas, se avivó el interés por su localización autóctona (Olagüe de Ros, 1995). Estas acciones fueron impulsadas por las autoridades gubernamentales y por los propios vacunadores, como fue el caso del propio Ruiz de Luzuriaga, tal y como se puede extraer del análisis de la correspondencia y documentación conservada sobre la introducción de la vacuna en España (Duro Torrijos, 2014).

Este es el resultado de mis observaciones, y las de mis amigos, aunque la yennerización ofrecerá cada día nuevas anomalías y nuevos resultados. He dirigido mis investigaciones por la península para saber si se conocía en alguna Provincia el yennerino en las vacas (Papeles sobre la vacuna, 1802, fol. 326).

Ruiz de Luzuriaga recopiló en su abultado epistolario varios ejemplos que lo atestiguan, como la carta fechada el 7 septiembre de 1801 remitida al secretario de la Academia desde Azpeitia, provincia de Guipúzcoa, por Javier de Murgartegui, indicándole que: “Nadie sabe nada por práctica, ni tampoco conocen a las vacas de por acá esta enfermedad, según me han dicho unos caseros a quienes se ha preguntado” (Papeles sobre la vacuna, 1801a, fol. 257).

Entre la correspondencia de Soria, el 9 de noviembre de 1801, el facultativo Josef Ubis informaba a Luzuriaga que:

En cuanto a las observaciones de si por aquí se rozan con el gabarro, y si por consiguiente ordeñan las vacas, hasta la presente nadie da razón de nada con fundamento, lo que ciertamente he preguntado y si pudiera adquirir algunos conocimientos, lo procuraré hacer con el mayor cuidado y con la brevedad que me sea posible, y aquí no hay tradición de nada en dicha materia (Papeles de la vacuna, 1801, fol. 214).

Desde Álava, el 26 de noviembre de 1801, por medio de una carta remitida por Manuel Álvarez Terrero, se le indicaba que a pesar de identificar pústulas similares en el ganado vacuno examinado no debe asociarse al ansiado *cowpox*.

Según los varios informes que he tomado, y en que van acorde estos naturales, es que no hay duda, en que a las vacas de cría les nacen muchas veces en las tetas postillas, por la mayor parte blancas y rojas, que las desmejoran mucho, y dan durante esta erupción menos leche.

Tengo entendido, y me han asegurado, que jamás se comunican a los que las ordeñan, por consiguiente, no pueden causar ninguno de los dolores que V me indica, y menos preservarles de las viruelas (Papeles sobre la vacuna, 1801b, fol. 116).

Estas investigaciones alcanzaron también a Cuba, como recoge la carta remitida desde La Habana el 19 de abril de 1802 por el destacado médico y provacunista Tomás Romay Chacón (1764-1849). Romay es definido como un filántropo laborioso y figura brillantísima de la cultura científica y social de la segunda mitad del siglo XVIII y primera del XIX, que dedicó encarecidamente a la lucha contra las enfermedades infectocontagiosas más representativas de la época, como la fiebre amarilla (López Sánchez, 1950). Pero la hazaña que lo inmortalizó fue la introducción y difusión de la vacuna jenneriana en Cuba, abandonando las comodidades de su hogar para marcharse al interior de la isla en busca del ansiado virus y de exponer la vida de sus propios hijos, a quienes vacunó como sujetos de prueba para vencer los temores del resto de la población (Diez Cabrera, Toledo Curbelo, 1997).

La carta comenzaba el texto, agradeciendo a Ruiz de Luzuriaga la mediación en su nombramiento como Socio Corresponsal de la Academia, para continuar detallando su destacada labor como introductor y difusor de la vacuna en la isla destacando, en este punto, las referencias obtenidas relativas a la posible localización de fluido vacuno autóctono, dato que se disponía a comprobar: “Tengo esperanzas de conseguirla en esta Isla, pues un vecino de un Pueblo interior, donde se cría mucho ganado vacuno, me asegura haber observado en las vacas esos granos, y que solían pegarlos a los que las ordeñaban; pero no advirtió ni sabía que se hubiese notado si a estas personas les habían dado” (Papeles de la vacuna, 1802, fol. 119).

El interés de Romay en la propagación y localización del fluido vacunal en la Isla queda patente en la publicación de una *Memoria sobre la introducción y progresos de la vacuna en la isla de Cuba* en 1805 (Romay, 1805). En ella, reconocía cómo los beneficios derivados de las vacunaciones realizadas en Europa y en los recién constituidos EEUU llegaron a la isla por medio de la prensa.

Romay y otros renombrados habaneros comenzaron la búsqueda del fluido vacunal. Para ello, se acordó junto con la Real Sociedad Económica de la Habana y la Junta Económica del Real Consulado otorgar un premio de trescientos pesos a quien condujese el virus vacuno de otros países y cuatrocientos pesos, a quien lo encontrase en nuestras vacas (Romay, 1805). La propia prensa periódica, el 3 de febrero de 1803, publicaba un artículo con la recompensa a quien encontrase vacas contagiadas en las haciendas locales (Hernández Aguilar, 2025).

Pero a pesar de que los dueños de las haciendas afirmaban la presencia del *cowpox* en las vacas de la región, nadie reclamó el premio como indica el propio Romay (1805).

Ese interés en la localización del fluido vacunal autóctono en el continente americano se evidenció también durante los prolegómenos de la Real Expedición Filantrópica de la Vacuna (1803-1810). Se discutieron varios proyectos, entre ellos el de Francisco Xavier Balmis (1753-1819), que resultó finalmente elegido y que será ampliamente tratado en otro capítulo del presente monográfico, y el proyecto de José Felipe Flores, datado el 28 de febrero de 1803, en el que se destacaba:

Los estragos que hacen las viruelas en América se recomienda mucho la inoculación de la vacuna, que no se ha puesto ya en ejecución en Guatemala por no haberse encontrado viruelas de las vacas, y haber llegado sin virtud el pus Vacuno conducido entre dos cristales a la Habana y Veracruz, de donde se había solicitado con instancia.

… Se buscaron sin descansar las viruelas de las vacas, se examinaban estos animales en todas partes, y no encontrando el virus, el doctor Córdoba se proponía experiencias originales, inoculando ovejas, fundadas en un analogismo perfecto… La inspección de las vacas se seguía, la inoculación de las ovejas se repetía y no se olvidaban los encargos por todas partes para solicitar el virus (Díaz de Yraola, 1948, p.127).

En este mismo contexto, se encuentran las diligencias tomadas por el propio virrey de Nuevo México, José Yturrigaray, quien formó una comisión, escasamente estudiada, bajo la dirección de Félix Garda y Ferraris, como certifica el escribano de la población de Toluca el 22 de marzo de 1803, al indicar que: “El Cirujano del Regimiento de Infantería de Nueva España, comisionado por la superioridad del Excmo. Señor Virrey…, ha realizado el reconocimiento en todas las Haciendas de la Jurisdicción para la inspección de la Viruela Vacuna” (Documentos …, 1803-1811, fol. 152).

Entre marzo y septiembre de 1803, el facultativo desarrolló su cometido, pero no logró encontrar vacas contagiadas y dudaba que el virus existiera en el territorio novohispano; por este motivo, solicita permiso para viajar a EEUU, siendo este desestimado a finales de septiembre por el propio virrey Iturrigaray (Hernández Aguilar, 2025).

Precisamente, a primeros de septiembre se comunicó la Real Cédula emitida por el monarca Carlos IV que anunciaba la organización de la expedición dirigida por Balmis para propagar la vacuna contra la viruela (Tuells, Duro Torrijos, 2013b; Hernández Aguilar, 2025).

Durante el desarrollo de la misma, Balmis y su ayudante Gutiérrez Robredo creyeron encontrarla durante su paso por tierras novohispanas, en el Valle de Atlixco y en Valladolid de Michoacán a finales de 1804. Aunque seis años más tarde el propio Balmis, tras ser nuevamente comisionado, reconoció su ausencia e incluso su engaño: “Descubrió que esto fue una superchería de cierto bribón, que por apoderarse de 12 pesos que ofreció de premio el señor obispo de Puebla a quien lo descubriese, lo introdujo a la misma vaca quien sabe por qué medios, pero con una constante y larga experiencia asienta que el dicho *cowpox* no le hay en las vacas de aquí” (Tuells, Duro Torrijos, 2013b, p.55).

El interés en la obtención y localización de fluido vacunal autóctono que emanó de las esferas gubernativas tal y como se recoge en el escrito por el secretario de Estado Pedro Ceballos y Guerra, dirigido al obispo de Orense el 6 de septiembre de 1803:

Siendo de la mayor importancia averiguar si en España hay vacas que tengan el fluido o suero vacuno de la misma índole que las de los condados de Gloucester, de las cuales se ha surtido hasta ahora la Europa, por no haberse encontrado en otra parte alguna, y habiendo tenido noticias que dan lugar a inferir con bastante probabilidad que las vacas de los valles de oro, y de Cuadramón, sitios en ese Obispado, dan entre otros un fluido vacuno semejante al que dan las de los referidos condados, observándose como en estos, que los que las ordeñan no padecen la viruela humana, si por alguna grieta de sus manos se les inocula espontáneamente la vacuna, el rey enterado de todo lo referido, me manda participarlo (Carta secretario..., 1803 fol. 252).

Esas aspiraciones no obtuvieron las noticias deseadas, pero denotan el interés e implicación institucional en la obtención y conservación del fluido vacunal. En este sentido, los primeros vacunadores, y ante la estéril búsqueda de la obtención autóctona del virus de viruela bovina, dependieron de una fuente externa de linfa, centrándose en el desarrollo tecnológico, en las técnicas médicas y herramientas para transportar el fluido vacunal de forma segura. Unos métodos que fueron adaptándose progresivamente tras fallar los primeros intentos de introducir el pus vacuno en lugares calurosos, donde el clima lo deterioraba y le restaba su capacidad infecciosa (Hernández Aguilar, 2025).

## Estrategias para el transporte y movilidad segura de la vacuna contra la viruela

A los intentos de localización del fluido vacunal de forma autóctona para garantizar el suministro del mismo debe añadirse el correcto método de obtención. Todo el éxito de las vacunaciones dependía directamente de la obtención de la materia vacunal.

En este contexto, proliferó el número de publicaciones de tratados y noticias periódicas que, siguiendo un didactismo científico, exponían los recursos e ilustraban las diferentes fases clínicas del grano o vesícula, indicando el momento correcto para realizar la operación de brazo a brazo. Un ejemplo de ello lo constituye el texto publicado en 1801, con enorme repercusión y difusión en la época, del médico Pedro Hernández (1801, p.41-42):

Como el buen suceso de la operación depende principalmente del fluido que se introduce bajo la epidermis, se hace preciso saber elegir el tiempo en que el dicho fluido tiene aquel grado de perfección y madurez propia y eficaz para comunicar la enfermedad. El tiempo más propicio es desde el séptimo al undécimo día de la enfermedad, porque antes de esta época, se halla la vejiguilla muy poco descubierta, y el fluido que tiene no está bastante elaborado, ni con perfección.

Superadas estas premisas, su conservación y los medios para transportar la vacuna constituyeron otro de los problemas esenciales que rodearon al fluido jenneriano.

Las grandes distancias que la vacuna debía realizar representaron un serio problema, ya que en muchas ocasiones el fluido llegaba seco e inservible, esencialmente en recorridos donde estaba expuesto a variaciones de temperaturas que aceleraban o retrasaban el proceso de calidad. La temperatura fue un elemento resaltado entre los facultativos de la época. Así, Joseph Morales, en su publicación *Cartilla de vacunas, de 1805*, destacaba que, a pesar de poder vacunarse en cualquier momento, hay que tener presente que “el calor adelanta algo los progresos de la vacuna, el frío algo los retarda” (Ramírez Martín, 1999, p.524).

Las vacunaciones se podían practicar con “el humor vacunal fresco, y con el seco” (Balmis, 1804, p.192). Para el primer supuesto, se obtenía directamente del picado de un grano vacunal en la lanceta que se empleaba en el mismo acto vacunal de un sujeto que debía estar presente. “Esta operación se llama vacunar de brazo a brazo” (p.193).

En el segundo de los casos, el fluido vacunal se debía conservar en diversos medios que facilitaban su transporte. Se recurría al empapado de hilas de algodón en la propia lanceta o en el empleo de vidrios que prensaban y conservaban el fluido vacunal.

Procedimientos bien descritos, en el caso de hilas de algodón, “el humor vacunal se seca en las hilas en términos que se desquebraja cuando se las llega a tocar, y expone al médico a que comunique una falsa vacuna, o a que no produzca efecto alguno, dejando en ambos casos al vacunado en el peligro mismo de poder padecer las viruelas” (Balmis, 1804, p.193).

Por medio de la lanceta,

el pus vacunal resecado sobre la punta de una lanceta se halla en dos estados muy distintos. 1º, por lo común suele oxidar la lanceta, padeciendo por consiguiente una descomposición que le hace mudar de naturaleza, y produce la falsa vacuna, del mismo modo que lo hace la hila empapada en el humor vacunal seco, o deja causar efecto alguno. 2º, si la lanceta no se oxida, adquiere una consistencia vítrea transparente y cortante por sus bordes, y cuando se le ha introducido dentro de la cutis, obra como un cuerpo extraño, causa una irritación muy pronta, y ocasiona la falsa vacuna (Balmis, 1804, p.193).

En el último supuesto, en el empleo de vidrios,

si el humor vacunal se coloca entre vidrios, puede conservar mucho tiempo sus propiedades, sin padecer alteración alguna, con tal que se observen las precauciones siguientes; luego que la vejiguilla circular del grano se ha picado en toda su extensión, se recoge en un vidrio chato y liso el fluido que se derrama y al instante, que contiene ya una cantidad proporcionada, se aplica otro vidrio de igual magnitud, y cuando ambos están igualmente cargados de pus se juntan los dos vidrios por las superficies humedecidas, cerrando exactamente sus bordes con un poco de lacre. Luego que estos vidrios han llegado al lugar destinado, se les despega fácilmente con arrimarles un carbón encendido, y en queriendo vacunar, se echa en uno de los dos vidrios una gota de agua destilada, removiéndola bien con la punta de la lanceta (Balmis, 1804, p.194).

Las dos primeras opciones, hilas de algodón y la propia lanceta, presentaban inconvenientes, no garantizándose la calidad del fluido y, por lo tanto, sus recursos profilácticos (Ramírez Martín, 1999, p.531).

La opción de los cristales constituía la modalidad más segura, de hecho, fue gracias a este procedimiento el modo con el que llegó a España y se extendió por ella.

La técnica fue recogida por múltiples tratados sobre la vacuna, e incluso por la propia prensa.

Se ha de esperar a que el grano o pústula la hay tomado todo su incremento, hágasele entonces una incisión circular para abrir la mayor parte que sea posible de las vejigas de que se compone, aplíquese una porción de hilas de un volumen igual a la concavidad del cristal del lado que estén más esponjadas y suaves y comprímanse de cuando en cuando sobre la pústula con la hoja de la lanceta, de esta manera basta una sola pústula para empaparlas bien. La ventaja de poder recibir fresco el fluido vacuno a cualquier distancia y conservarlo en caso de interrupción en las vacunaciones es muy importante (Carta sobre..., 9 sept. 1802, p.174.).

Para usar el fluido vacuno conservado por este procedimiento se debía mojar el instrumento de aplicación con agua fría y “bien clara”, y con él se frotaba la superficie donde se localiza el fluido hasta que adquiriese una consistencia ligeramente espesa pasando a continuación a “cargar o mojar” las lancetas con las que se han de practicar las picaduras (Calleja, 1814, p.5-6).

La necesidad de recorrer grandes distancias para propagar el fluido vacunal hizo que se experimentase con otros métodos, como la inserción en un sujeto sano de material procedente de las costras pulverizadas resultantes de un sujeto enfermo de viruela.

La prensa canalizó todos los resultados y avances referentes a la vacuna contra la viruela producidos en Europa, muchas de estas crónicas son firmadas por destacados activistas provacunistas, como Jean de Carro, médico suizo y uno de los introductores de la vacunación en Centroeuropa y Oriente Próximo.

Se ha creído que la costra del grano vacuno no tenía la virtud de reproducir otro grano y en este punto hallábamos yo y otros una diferencia entre las viruelas y la vacuna, pero un cirujano de Edimburgo llamado Bryce ha repetido gran número de experimentos que prueban que dicha costra pulverizada y humedecida con agua produce la vacuna con tanta felicidad como el fluido más cristalino. Este hecho me parece de la mayor importancia por la facilidad que presenta conservarlo mucho tiempo el virus vacuno en las costras y de enviarlo a largas distancias. Viena 2 de febrero de 1804. De Carro (Nuevo…, 10 mayo 1804, p.304).

Continuando las palabras de Jean de Carro respecto a la importancia de obtener el fluido vacuno fresco en cualquier distancia y conservarlo en caso de interrumpirse las vacunaciones, en un claro ejemplo de innovación y desarrollo tecnológico, el mismo periódico resaltaba la elaboración de unos frasquitos diseñados en Londres a finales de 1800, por el Sir Lewis, boticario de la institución de la vacuna (Duncan, 1801).

Ahora se acaba de discurrir en Inglaterra un medio sencillo de transportar el fluido vacuno, que les sale muy bien en la práctica. Como lo principal es impedir que esté expuesto al aire, lo recogen en un hoyito liso hecho en la extremidad de un tapón de cristal de un frasquito de lo mismo. Este tapón se alarga hasta llegar casi al fondo del frasquito, y lo hacen tan bien ajustado a la boca que cierra con la mayor exactitud. De aquí es que el fluido vacuno, que no está en contacto sino con aquella cantidad corta de aire que contiene el estrecho frasquito, no se llega a descomponer ni evaporar. Cuando se quiere hacer uso de él, basta abrir el pomito y pasar muchas veces una lanceta, que se mete ante en agua fría, sobre la pequeña concavidad que está a la extremidad del tapón. El que hace estos frascos tiene privilegio exclusivo y los vende en Londres en casa de Parker, Fleet Street (Carta sobre…, 9 sept. 1802, p.176).

Fue, precisamente, el responsable de la publicación del periódico, Juan Antonio Melón González (1758-1843), clérigo, erudito y escritor ilustrado, quien remitió una carta fechada el 10 de agosto de 1803 al propio secretario de Estado y del Despacho, Pedro Cevallos y Guerra (1759-1839), para indicarle la importancia de la noticia y la necesidad de ponerla en práctica en nuestro país.

Esta carta coincidía con los preparativos de la Real Expedición Filantrópica de la Vacuna, expedición cuyo objetivo primordial versaba en propagar la vacuna contra la viruela en las colonias de ultramar, para ello recurriendo al empleo de niños como reservorio del fluido, método que el propio Melón cuestionaba:

Se dice que la expedición que va de orden del Rey a propagar la vacuna en América llevará individuos en que irla transmitiendo sucesivamente para conservarla fresca: este método está expuesto a contingencias que pueden frustrar el objeto de tan útil empresa, como, por ejemplo, si enfermase o muriese el que se hallaba vacunado, si en alguno no prendiese la vacuna, como sucede a veces, ó si le saliese falsa como también se suele ver. Para evitar estos inconvenientes han discurrido nuevamente en Inglaterra un medio de transportar el fluido vacuno en frasquitos a propósito, que se venden en Londres en casa de Parker Fleet Street, los que han merecido tanto la aprobación de aquellos facultativos, que ha concedido el rey privilegio exclusivo al autor de ellos (Carta secretario..., 1803, fol. 258).

Ante tales referencias, Melón le recomendó a Cevallos que el secretario de la embajada en Londres, José de Anduaga y Garimberti (1751-1822), remitiera en el primer correo unos frasquitos para poder producirlos en la fábrica de vidrio de San Ildefonso, recipientes que garantizarían la conservación del fluido vacunal en la expedición que iba a partir a América.

Yo sé que no los tienen los que de orden del rey nuestro señor pasan a América, y acaso será del agrado de S.M., que V.E., prevenga al señor Anduaga, que con el primer correo remita una o dos docenas, con el método de recoger en ellos el fluido, para que a su vista se puedan mandar hacer en San Ildefonso, y proveer no solo a los que van a América, sino al público que quiera usar de ellos para propagar tan importante preservativo (Carta secretario..., 1803, fol. 259).

Una nota marginal en el documenta recoge como se ordena la solicitud: “Pídanse a Anduaga las dos docenas de frasquitos con el método de recoger en ellos el fluido” (Carta secretario..., 1803, fol. 259).

En cumplimiento de la Real Orden, Anduaga, remitió el 3 de septiembre a vuelta de correo desde Londres las demandas junto con un manual para su empleo.

Remito con el correo de gabinete D. Eugenio Gutiérrez en un paquete dos docenas de frasquitos de cristal comprados en casa de Parker en Fleet Street que sirven para transportar y conservar el fluido vacuno. Me han asegurado que no hay, ni se necesita más explicación acerca del uso de estos frasquitos que el saber, que por la figura que forma tapón y la mucha extensión de la parte que entra dentro del frasco tienen la ventaja de cerrarse herméticamente, y la de que pegándose el fluido en la parte interior del tapón, sirve este para extraer cómodamente la porción que convenga con sólo sacar el tapón (Carta secretario..., 1803, fol. 264).

Una vez recibidos los novedosos frasquitos, José Antonio Caballero (1754-1821), secretario de Estado y del despacho de Gracia y Justicia, remitió a la Junta de Cirujanos de Cámara del Rey 12 de los 24 recipientes para ser examinados y “si mereciesen aquí la aceptación y aprobación que han obtenido estos frascos en Inglaterra, se podrán similar fácilmente en la real fábrica de San Ildefonso” (Carta secretario..., 1803, fol. 264).

Quienes dictaminaran que:

Han parecido muy útiles y preferibles a los vidrios usados; porque, siendo necesario más tiempo para colocar en uno y otro de estos el fluido, se expone a volatizarse o disiparse con el contacto del aire, al paso que es muy pronta la acción de ponerla en los frasquitos, por consiguiente, más segura su preservación, y más fácil y cómodo el transporte (Carta secretario..., 1803, fol. 252).

Para asegurar tal afirmación la Junta decidió enviar seis de los doce frasquitos al Colegio de San Carlos de Madrid para que procedieran a la recopilación de fluido vacunal

de verdaderos vacunados y pongan en tres [frascos] el fluido solo y, en los otros, cantidad proporcionada disuelta en agua destilada, por haberse experimentado anteriormente que, disuelto así, el pus de la viruela natural producía el mismo y más benigno efecto que ingiriéndolo puro; y si sucede lo propio con el vacuno, sería más expedita la propagación de la vacuna, por la mayor cantidad de fluido, que podrá remitirse en cada frasquito (Carta secretario..., 1803, fol. 252).

En el mismo texto, fechado el 21 de octubre de 1803, se dio la conformidad para la fabricación de una porción de frasquitos en la Real Fábrica de San Ildefonso con la finalidad de extender su uso.

El interés mostrado desde las instituciones gubernativas por la correcta conservación del fluido vacunal con la finalidad de garantizar su transporte fue tan elevado, que, el 29 de noviembre de ese mismo año, Anduaga volvió a remitir otro frasquito desde Londres, esta vez con la particularidad que en el recipiente ya portaba el fluido vacunal según el método adoptado por el propio centro emisor: “Al frasquito acompaña una hoja de papel impresa en que el instituto indica las reglas para la inoculación de la vacuna; y además un tomito en octavo que comprende el informe o relación sobre la inoculación de la vacuna según la práctica del mismo instituto en los años 1800, 801 y 802” (Carta secretario..., 1803, fol. 268).

Ruiz de Luzuriaga, como secretario de la Academia Médica madrileña, recibió, en carta del 11 de febrero de 1804 remitida por el propio Cevallos, “un frasquito de los que se han inventado últimamente en Inglaterra ... en el que va colocado el virus mismo”, junto con unas instrucciones “a fin de que la Academia Médica haga de todo el uso que estime conveniente” (Carta de Pedro Ceballos…, 1804).

Tan solo cinco días más tarde, el propio secretario de la academia lo exponía en la sesión ordinaria de la institución:

El Secretario leyó el acuerdo de la Junta anterior y dio cuenta de una orden de S.M., comunicada por el Excmo. Sr. Protector con fecha de 11 de Febrero remitiendo un frasquito inventado últimamente en Inglaterra para conservar el fluido vacunal según el método adoptado por el instituto que promueve la vacunación en Londres, con un tomito inglés que comprende el informe sobre la vacuna según práctica del mismo instituto en 1800, 801 y 802, y una hoja suelta en que se indican las reglas para dicha operación. La Academia acordó se pasase a los señores [Juan de] Peñalver y [Tomás García] suelto a fin de que le incluyan en el informe de la vacunación de que están encargados (Libro de actas…, 1804, fol. 321).

Pero las grandes expectativas puestas en tan útil recipiente, “de forma que comprobada la utilidad podría fabricarse porción competente y ponerse de venta en el Real Almacén” (Libro de actas…, 1804, fol. 322), comenzaron a diluirse como le trasladó Cevallos a Caballero desde la Secretaria de Estado y del Despacho, el 13 de abril, al indicarle que “no habiendo correspondido los efectos experimentados en siete personas distintas a las esperanzas de la Junta” (Carta de Pedro Ceballos…, 1804), refiriéndose a la Junta de Cirujanos de Cámara, se

ha propuesto y determinado el rey que su embajador en Londres pida al doctor Jenner otra instrucción del modo de recoger el fluido y colocarlo en los frasquitos, y si se toma inmediatamente de las vacas o de los vacunados, encargando al mismo embajador que remita con la instrucción dos frasquitos con el fluido colocado según ella, y expresando el método, que se observa en Inglaterra (Carta de Pedro Ceballos…, 1804).

Las expectativas iníciales depositadas en los frasquitos ingleses se vieron truncadas ante la escasez de resultados. Siendo la misma persona que se interesó por su práctica en la península, el responsable del *Semanario de Agricultura y Artes Dirigido a Párrocos*, Juan Antonio Melón, quién volvería a publicar, el 12 de abril de 1804, en el mismo rotativo, la falta de practicidad y fiabilidad del recipiente.

Como los ingleses habían alabado el método de conducir el fluido vacuno en unos frasquitos hechos a propósito, pidió a Londres con un celo muy plausible el señor ministro de Estado una porción de ellos, que probados en Madrid se ha visto que no lo conservan bien. Dos cosas pueden contribuir a ello, la primera, que el tapón de dichos frasquillos no se prolongan bastante hasta llenar todo su hueco, quedando en ellos, después de cerrados, suficiente cantidad de aire para alterar el fluido; y la segunda que, siendo los frasquillos de cristal, pudo la luz tener acción sobre el mismo fluido y privarle de su virtud (Concluye…, 12 abr. 1804, p.239).

Un mes más tarde, el 12 de mayo, Ruiz de Luzuriaga ratificó esta misma concepción, trasladando al ministro Caballero el resultado de las experiencias prácticas con la muestra de fluido vacunal remitida en el novedoso recipiente a la academia médica el 11 de febrero,

habiendo practicado el cirujano D. Juan de Azaola varias vacunaciones con él, han fallado todas, lo que proviene sin duda de haberse abierto el frasquito antes de llegar a nuestras manos, y disiparse el gas hidrógeno con que debe llenarse el frasquito, método muy engorroso y nada preferible al de los cristales en que se ha conservado perfectamente en todas las remesas que he hecho en toda la península, aunque no surtieron las que hice a la Habana (Carta de Pedro Ceballos…, 1804).

Como podemos observar, el mismo Ruiz de Luzuriaga confirmó los resultados negativos emitidos desde la Junta de Cirujanos de Cámara, defendiendo el método de conservación empleado hasta el momento, el uso de los cristales sobre los que él mismo vertebró su red de corresponsales, experiencias con excelentes resultados a excepción de la remesa enviada a Tomás Romay en la Habana.

Por lo tanto, en estos primeros pasos de la introducción de la vacuna son identificables tres métodos de conservación y transporte del fluido vacunal. En seco, con la impregnación de hilos de seda o algodón con el pus vacuno, secado y almacenado; en vidrio, mediante la conservación y envío del pus vacuna sellado entre láminas de cristal o dentro de frascos. No obstante, se comprobó que ambos métodos eran inadecuados para travesías largas y, en especial, para los climas cálidos, pues el pus se deterioraba y perdía su capacidad infecciosa. Por lo tanto, la opción denominada *in vivo* o de brazo a brazo, se constituyó como el método más seguro de propagar la vacuna, una técnica que requería de una logística, infraestructura sanitaria y de esfuerzos institucionales (Hernández Aguilar, 2025). Un claro ejemplo lo constituye la preparación y desarrollo de la Real Expedición Filantrópica de la Vacuna, que pasamos a detallar.

Las inquietudes por conseguir transportar el fluido, como podemos observar, estaban presentes y aún más si cabe en el caso de los territorios de ultramar donde la viruela seguía muy presente. Estas circunstancias movieron a las autoridades de los distintos virreinatos a solicitar a la Corona que enviase cuanto antes el nuevo remedio. La monarquía borbónica, que había introducido las expediciones científicas como elemento de progreso y dominio, aprobó el proyecto conocido como Real Expedición Filantrópica de la Vacuna, al mando de Francisco Javier Balmis, comisión que se decantó para ejecutarla por reservorios humano, desde los cuales se transferiría la vacuna de brazo a brazo, construyéndose una cadena necesaria cuyos eslabones los configurarían múltiples niños obtenidos en su mayoría de inclusas y orfanatos.

La configuración, existencia y preparación de la Expedición de la vacuna fue difundida en el mes de julio de 1803 en la prensa periódica, como el recogido por el *Mercurio de España:*

El precioso descubrimiento de la vacuna, acreditado en España, y casi en toda Europa, como un preservativo eficaz de las viruelas naturales, ha excitado la paternal solicitud del rey a propagarlo en sus dominios de Indias, donde suele ser mayor el número de víctimas que sacrifica esta horrorosa plaga. Con tal objeto se ha servido mandar S.M., después de oído el dictamen del Consejo y de algunos sabios, que se forme una expedición marítima, compuesta de facultativos hábiles y adictos a la empresa, dirigido por el médico honorario de Cámara D. Francisco Xavier de Balmis; y costeada de su Real erario; los cuales sin perder gastos ni fatigas lleven suficiente número de niños a quienes inoculen sucesivamente en el curso de la navegación, y conservando por este y otros medios el fluido vacunal en toda su eficacia, hagan a su arribo las primeras operaciones de brazo a brazo, las que continuarán después en ambas Américas y si fuese dable en las islas Filipinas (Noticia de la expedición…, jul. 1803, p.382).

La propuesta de Balmis estaba basada en la utilización exclusiva de niños para realizar el transporte del fluido. Era un procedimiento recomendado por el propio Jenner (Rusnock, 2009). Desde ese momento, los niños cobraron un valor sustancial que quedó expuesto en el Reglamento y Derrotero presentado por Balmis a la Junta de Cirujanos (Castillo y Domper, 1912). Recibieron el nombre de niños vacuníferos aquellos escarificados por el *cowpox* de los que se podría obtener fluido vacuno transcurridos entre ocho y diez días, periodo en el que el grano estaba maduro. A partir de un primer niño se inmunizaría a otro, estableciéndose una cadena de inoculaciones sucesivas, que permitiría el transporte de la vacuna.

Balmis recomendaba la recolecta de niños de entre 5 y 8 años que no hubieran padecido la viruela, lo que suponía una cierta garantía frente a los adultos que sí podían haberla padecido. Por eso, puso especial énfasis en condicionar la elección a que se “averigüe con escrupulosidad, que nos asegure de que aún no han padecido las viruelas naturales, ni las inoculadas, y tampoco que no han sido vacunados: porque todos estos son inútiles” (Ramírez Martínez, 2003, p.86). Para garantizar el procedimiento y evitar fracasos, inoculaba a dos niños sucesivamente con punciones múltiples, lo que permitía obtener varios granos vacunales en cada uno de ellos. El niño vacunífero estuvo presente en todas las rutas y etapas de la Real Expedición Filantrópica de la Vacuna.

La utilización del niño con fines científicos fue una realidad durante todo el siglo XVIII, con un sentido utilitarista y de generalización del método científico al servicio e interés del Estado. La Real Expedición Filantrópica de la Vacuna fue un claro ejemplo de esta utilización, ya que el éxito de la misión dependía directamente del número de niños, su continua provisión para mantener la cadena brazo a brazo y su mantenimiento en perfecto estado de salud.

## Consideraciones finales

La conservación y transportes del fluido vacunal, en los inicios de la vacunación contra la viruela, implicaron desafíos técnicos que determinaron su eficacia. Los métodos de conservación utilizados, desde la conservación entre cristales y frascos experimentales hasta su propagación mediante la vacunación brazo a brazo, revelaron limitaciones específicas en condiciones climatológicas adversas y largas travesías, lo que impulsó innovaciones logísticas determinantes para la expansión efectiva de la vacuna.

Asimismo, es relevante el protagonismo de las redes institucionales, la prensa periódica y las innovaciones como vector de la vacunación, cuya interacción posibilitó la transmisión transatlántica del fluido vacunal. Esta perspectiva contribuye a un enfoque historiográfico de la materialidad y las redes tecnológicas que condicionaron la salud pública temprana.

El alcance o éxito de las dichas campañas de vacunación no estuvo exento de críticas. De hecho, fue una práctica de prevención que suscitó incertidumbre, miedo o rechazo. Será precisamente las reacciones sociales y actuación de las autoridades gubernamentales objeto de análisis desde distintas entidades locales cuyos aportes constituyen una gran contribución a la historia de la salud pública (Molina del Villar, 2017).

Desde una perspectiva tecnológica y científica, la vacunación contra la viruela impulsó el desarrollo de nuevos métodos de conservación y transporte de vacunas. El interés por mejorar la estabilidad del fluido vacunal llevó a la experimentación con diferentes técnicas, como la inserción de la vacuna en hilos de algodón, su almacenamiento en cristales o el uso de frascos sellados herméticamente. Aunque algunas de estas técnicas presentaron limitaciones, sentaron las bases para el desarrollo de métodos más sofisticados en el futuro

De este modo, la obtención y conservación del fluido vacunal, constituyó un desafío técnico crucial para garantizar la efectividad de la vacunación, un reto que requería de una correcta organización e implicación de las esferas gubernamentales y científicas.

En este sentido, y a modo de conclusión, es destacable la opinión expuesta por el secretario de la Academia de Medicina de Madrid, Ruiz de Luzuriaga, en diciembre de 1803, donde resaltaba la necesidad de implementar una vacunación metódica con la creación de una comisión o juntas centrales, siguiendo los ejemplos de Francia e Inglaterra, establecimientos encargados de supervisar el seguimiento de los casos e impedir situaciones de desabastecimiento del fluido vacunal.

A beneficio de este establecimiento filantrópico se exonera al gobierno de tener como en España que atender a todos los objetos de beneficencia pública, y una comisión central establecida en la capital con los personajes más distinguidos, que correspondiesen con los capitanes generales, arzobispos, obispos y los médicos y cirujanos de la mayor reputación y mérito, y una botica para surtir de Extracto de Saturno o algún medicamento poco costoso, que pueda ocurrir necesitarse rara vez en las vacunaciones. Así se podría mantener el fluido vacuno en todo el Reino, evitar la introducción de la falsa vacuna y promover el aumento de la población.

Un taller establecido en la fábrica de cristales para aprovechar todos los cristales rotos en que se remitiese el fluido vacuno, cierta franquicia en los correos cuando se remita este fluido sellado con el sello de la comisión, y un poco de protección de parte del gobierno serían los verdaderos medios de que desde las capitales se difundiese al menor lugarejo este saludable y benéfico descubrimiento, siendo muy dificultoso que llegase a faltar a un tiempo en todas partes, y previniendo que se franqueé así gratuitamente de unas comisiones a otras, teniendo formadas unas instrucciones en forma de catecismo acerca del método de practicar las vacunaciones y de evitar las falsas vacunas que no conducirían a preservar de las viruelas (Informe…, dic. 1803, fol. 4).

Surge, así, un interés sanitario enmarcado en un contexto que se caracteriza por la instauración de una nueva práctica gubernamental, centrado sobre el conjunto de los seres vivos organizados como población.

La consideración de la salud como uno de los objetivos básicos del poder político fue una inquietud recurrente del reformismo ilustrado europeo, que trató de alcanzarse mediante el despliegue de una política sanitaria cuyos caracteres esenciales fueron la adopción de medidas de control de la mortalidad, la infancia y natalidad, en definitiva, el funcionamiento de la medicina como instrumento de control social.

Una ciencia renovada que, para alcanzar a todos los estrados sociales, contó con plataformas como la prensa periódica y las publicaciones científicas, ambas con funciones médicas y educativas en la medicalización del núcleo familiar.

Pero a pesar de las inquietudes y expresiones en pro de la implementación de la vacunación, la respuesta gubernamental fue tibia, no se promulgó ninguna medida legislativa que arropase el invento de Jenner. Una excepción fue la emisión de las Reales Ordenes, entre los meses de agosto y septiembre de 1803, en las que se comunicaba la Expedición Filantrópica de la Vacuna y la Real Cédula de 1805, por la que se promulgó la instalación de salas de vacunación en los hospitales de las capitales de provincia, en las que se debía desarrollar esta práctica de forma gratuita para los pobres y que contó con una escasa aceptación entre la población.

Finalmente, la historia de la vacunación contra la viruela muestra la complejidad sociopolítica y técnica inherente a la adopción de innovaciones biomédicas. Más allá de los avances científicos y tecnológicos, la aceptación de la vacunación requirió de esfuerzos educativos, estrategias de comunicación y el respaldo de las autoridades. Unos desafíos que enfrentaron los primeros vacunadores que reflejan problemáticas que persisten en la actualidad, como la resistencia a las vacunas, la desinformación y las barreras logísticas en la distribución de inmunizaciones.

## Data Availability

No están en repositorio.
